# Being in the Moment So You Can Keep Moving Forward: Mindfulness and Rumination Mediate the Relationship between Attachment Orientations and Negative Conflict Styles

**DOI:** 10.3390/ijerph17186472

**Published:** 2020-09-05

**Authors:** Rachael E. Quickert, Tara K. MacDonald

**Affiliations:** Department of Psychology, Queen’s University, Kingston, ON K7L 3L3, Canada; tmacdon@queensu.ca

**Keywords:** attachment orientations, mindfulness, rumination, experiential avoidance, conflict styles

## Abstract

Attachment insecurity has been associated with negative behaviors during conflict and decreased relationship satisfaction. We theorize that individuals high in attachment anxiety and/or avoidance are less mindful during conflict with their romantic partners, and thus more likely to ruminate. Decreased mindfulness and higher levels of rumination may be important mechanisms in the relationship between attachment insecurity and conflict behavior, as it may be more difficult to engage in constructive problem-solving skills when one is distracted from the present moment. We conducted an online survey assessing 360 participants’ attachment orientations, levels of mindfulness and rumination, behavior during conflict, and experience with mindfulness activities. Using a serial mediation model, we found that mindfulness and rumination mediated the relationship between attachment insecurity and negative conflict behaviors. We further discovered that individuals high in attachment insecurity were more likely to report negative experiences with mindfulness activities (i.e., meditation and yoga), and that this relationship was mediated by higher levels of experiential avoidance, or a fear of engaging with one’s own thoughts and feelings. We discuss the importance of increasing mindfulness and decreasing both rumination and experiential avoidance to assist individuals high in attachment insecurity in navigating relationship conflict using more constructive and relationship-promoting strategies.

## 1. Introduction

### 1.1. Attachment Theory

Human interaction is a crucial part of peoples’ lives and influences how we see ourselves and the world [1]. Early interactions with primary caregivers create expectations for relationships throughout the lifespan [2,3]. Attachment theory posits that children create “working models” of expectations in relationships (i.e., whether others can be relied upon during difficult times). These working models are developed and refined over time by experiences in close relationships and influence how individuals perceive and react to their adult romantic partners as well [4].

Attachment in adulthood is conceptualized across two continuous dimensions: attachment anxiety and attachment avoidance [5]. Individuals high in attachment anxiety fear rejection and/or abandonment by romantic partners, whereas individuals high in attachment avoidance are uncomfortable with emotional intimacy and/or closeness [5]. Individuals high in either dimension of attachment (also referred to as “attachment insecurity”) are more likely to experience strong emotional and behavioral reactions to conflict in relationships. Individuals high in attachment anxiety are more likely to engage in “hyperactivating strategies” with the goal of maintaining proximity to their partners (e.g., engaging in excessive reassurance seeking to assess if one’s partner still loves him/her). Individuals high in attachment avoidance are more likely to engage in “deactivating strategies” with the goal of maintaining distance and autonomy from their partners (e.g., increasing physical or emotional space from one’s partner; see descriptions of these processes in references [6,7,8]).

Individuals can be high in either dimension of attachment (i.e., attachment anxiety or avoidance), or high on both dimensions at the same time (e.g., fearing rejection and simultaneously having difficulties with closeness). In fact, attachment anxiety and avoidance are moderately correlated. A meta-analysis of 100 studies of attachment anxiety and avoidance reported an average correlation coefficient of *r* = 0.32 [9]. Attachment behaviors are most evident during conflict or stress, as conflict and stress are theorized to activate working models of attachment [3,10].

A large literature base supports the association between being high on either dimension of attachment and negative individual (e.g., references [6,8,11,12]) and interpersonal outcomes (e.g., references [6,8,13]). Specifically, attachment insecurity has been related to less constructive conflict management styles [14,15,16,17] and reduced satisfaction in relationships (see Reference [18] for a meta-analysis).

One specific cognitive process related to attachment insecurity and interpersonal difficulties is rumination. Individuals high in attachment anxiety are more likely to endorse engaging in rumination [19,20]. Rumination is defined as repetitively thinking about events in the past, present, or future, which is often “unproductive,” and theorized to get in the way of conscious decision-making [20]. Individuals can become “hyper-focused” on aspects of an interpersonal interaction, and potentially miss other perspectives or experiences in the present moment [21]. Rumination is associated with negative emotional experiences in relationships [19], challenges resolving conflict [22], and increased rates of emotional distress and psychopathology [21]. We argue that as conflict in romantic relationships activates working models of insecure attachment, individuals struggle to stay present and mindful during interactions with their partner, thus relating to rumination and more negative behavior during conflict.

### 1.2. Mindfulness

Mindfulness is broadly defined as paying attention to the present moment [23], reducing bias, judgment, and attachment to what is noticed [24]. Mindfulness practices have been present for thousands of years and are an important aspect of many religious and cultural traditions. In the past thirty years, there has been growing attention in the field of psychology examining the benefits of mindfulness for psychological well-being. Mindfulness practices have been associated with benefits for coping with stress, anxiety, and depression [23,25,26,27], improving compassion and increasing prosocial (e.g., helping or relationship-promoting) behaviors [28,29,30], bolstering attention and working (e.g., short-term) memory [31,32], improving self-regulatory abilities (e.g., self-control) [33], and supporting physical health [34,35,36]. Several papers suggest that the positive effects of mindfulness may occur through increased attention control, emotion regulation abilities, and self-awareness, which are associated with improved self-regulation skills [37,38,39,40,41].

With many reported benefits of mindfulness, clinical interventions have been created to teach this skill, such as Mindfulness-Based Stress Reduction [42,43], Mindfulness-Based Cognitive Therapy [44], Acceptance and Commitment Therapy [45], and Dialectical Behavior Therapy [46]. A large body of research supports the benefits of mindfulness interventions for improving mood, emotion regulation abilities, physical health, and interpersonal relationships (e.g., references [23,47,48,49,50,51]).

In the domain of romantic relationships, research has supported the benefits of mindfulness for couple satisfaction and relationship functioning [52]. We posit that paying attention to the present and reducing judgment would be beneficial for appreciating positive experiences in relationships, and for navigating interpersonal conflict using constructive and prosocial (i.e., relationship-promoting) tactics [24]. Further, improving skills in attention control, emotion regulation, and self-awareness (mechanisms suggested by reference [37]) may also predict longer and more satisfying relationships.

### 1.3. Mindfulness and Attachment Orientations

There is a large literature base supporting the benefits of mindfulness for individual and interpersonal wellbeing, and thus we reason that mindfulness may be a helpful tool for individuals high in attachment insecurity coping with stress in romantic relationships. Engaging in mindfulness may reduce the extremity of emotional and behavioral responses to conflict typical of individuals high in attachment anxiety and avoidance, as many of their difficulties may involve being detached from the present moment. Individuals high in attachment insecurity who perceive relational threat may struggle to remain in the present with their partners due to strong emotional reactions, and ruminating about the past or future [6,8]. These individuals may also experience difficulties detaching from personal biases and judgment (i.e., forming negative interpretations of ambiguous information [6,8,53]). Mindfulness may help individuals high in attachment insecurity manage their emotional reactions, reduce rumination, and engage in less biased processing of their partner’s behavior. In this way, mindfulness may help them to acknowledge their maladaptive behaviors, engage in coping, and employ more constructive strategies to navigate conflict. Indeed, researchers have discussed the potential utility of mindfulness as an intervention for individuals high in attachment anxiety or avoidance to manage emotion regulation difficulties, stress, anxious and depressive symptoms, and to improve wellbeing and relationship stability [54,55,56,57,58,59,60].

Much research supports the association between attachment orientations and lower levels of mindfulness [61,62,63,64,65,66,67,68]. For example, in one sample (70 primarily American adults on a meditation retreat), attachment anxiety and attachment avoidance could explain 42% of the variance in mindfulness [67]. Several studies report mechanisms in the relationship between attachment anxiety and lower levels of mindfulness: rumination, challenges with attentional control, and thought suppression [69,70]. These mechanisms were found to statistically explain variance in the relationship between attachment anxiety and mindfulness and are thus theorized to be part of the process underlying the relationship between these two variables. Meditation appears to be more challenging for individuals high in attachment insecurity due to higher levels of ruminative thoughts distracting them from the present moment, and a greater degree of challenge in detaching from these thoughts and controlling their attention to return to the present moment. It is important to note that these relationships among attachment orientations, rumination, and mindfulness appear to be bidirectional [68]. Altering levels of mindfulness has also been associated with changes in rumination [71].

Aside from rumination, there are other factors that make mindfulness more challenging for individuals high in attachment insecurity. The process of meditating involves paying attention and experiencing all thoughts and feelings, even when they are negative, which can be difficult even for the most skilled mindfulness practitioners [72,73]. If someone is especially averse to experiencing negative thoughts and feelings (i.e., someone low in distress tolerance or high in experiential avoidance [74,75]), that person would likely report greater challenges with being mindful in the presence of negative emotions. Distress tolerance represents the ability to withstand negative thoughts, feelings, and experiences. Experiential avoidance is a related, but more general construct, capturing individuals’ fear of engaging with thoughts, feelings, and experiences, and related avoidance behaviors. For someone high in these traits, it would be especially uncomfortable to focus on internal states when stress is present. The added awareness of one’s stress response associated with mindfulness may lead to catastrophization and exacerbated reactions to stress, rather than helping individuals cope with it. Attachment insecurity is indeed correlated with higher levels of experiential avoidance [76,77], and lower levels of distress tolerance [75], suggesting that present-moment awareness of negative thoughts and feelings would be especially challenging.

Overall, it seems that mindfulness is generally positive for relationships, but that individuals high in attachment insecurity struggle to be mindful and present in their relationships. This lack of mindfulness may be a mechanism that explains part of the relationship between attachment insecurity and negative outcomes in romantic relationships, especially during periods of stress (i.e., when attachment behaviors are most activated). Surveying the literature, it seems that rumination, low distress tolerance, and high experiential avoidance may all be implicated in the relationship between attachment insecurity and lower levels of mindfulness.

### 1.4. Present Study

We were interested in extending the literature on attachment and mindfulness to examine relations to specific behavior during conflict in romantic relationships. We assessed whether mindfulness and rumination mediate the link between attachment insecurity and conflict styles using the Romantic Partner Conflict Scale (RPCS, [78]). This scale is a self-report examining six of the most common behaviors exhibited during conflict: compromise, avoidance, interactional reactivity (i.e., high emotionality and expressiveness), separation, domination, and submission. Attachment anxiety and avoidance have been related to many negative conflict behaviors [14,15,16,17], and thus we hypothesized negative associations with compromise, and positive associations with avoidance, interactional reactivity, separation, and ironically, both domination and submission (see Reference [16] for an explanation of the link to both domination and submission).

**Figure 1:**
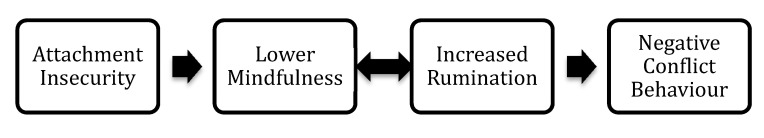
Hypothesized serial mediation model predicting the relationship between attachment insecurity (i.e., high anxiety and/or avoidance) and negative conflict behavior (i.e., lower compromise, increased interactional reactivity, etc.). Note that both orders of the two mediating variables were tested as the relationship between mindfulness and rumination is likely bidirectional.

We theorized that mindfulness and rumination would mediate the relationship between attachment insecurity and these negative conflict behaviors (i.e., be mechanisms in this relationship, statistically explain variance in this relationship). We predicted that individuals high in attachment anxiety or avoidance are likely to be less mindful during conflict with their partners, which would be associated with greater levels of rumination, and thus more negative conflict behaviors. Supported by past literature, attachment insecurity is associated with lower levels of mindfulness [61,62,63,64,65,66,67,68]. Being less attentive to the present moment, individuals high in attachment insecurity may be more likely to engage in rumination, repetitively thinking about events in the past, present, or future (e.g., past experiences of feeling abandoned by others, or worries about breaking up and future negative outcomes). Attachment insecurity is related to rumination [19,20], and we theorize that should be especially true for individuals who struggle to remain in the present moment due to low levels of mindfulness. Thus, we predict that with lower mindfulness and increased rumination, individuals high in attachment insecurity are likely to be less present and attuned to their partner, thus predicting less constructive conflict styles (e.g., less compromise, more interactional reactivity). We hypothesized a serial mediation from attachment insecurity to conflict styles, mediated by both mindfulness and rumination. As the relationship between mindfulness and rumination is likely bidirectional (i.e., low mindfulness may predict increased rumination, and increased rumination may predict even lower levels of mindfulness), we examined both orders of these serial mediating variables in separate statistical models (see hypothesis depicted visually in Figure 1).

As attachment insecurity is generally related to decreased relationship satisfaction (see Reference [18] for a meta-analysis), we hypothesized a similar serial mediation model linking attachment anxiety and avoidance to lower satisfaction in relationships, mediated by mindfulness and rumination. Being less mindful and ruminating to a greater extent may predict both more negative conflict behaviors, but also lower satisfaction in romantic relationships. If one is less focused on the present moment (due to both lower levels of mindfulness and increased rumination about the past and future), they are likely to be less focused on their partner. Less attention to the present moment may predict less satisfaction with one’s romantic relationship. Presence and active listening in romantic relationships have been associated with better coping during conflict and increased satisfaction [79].

**Figure 2:**
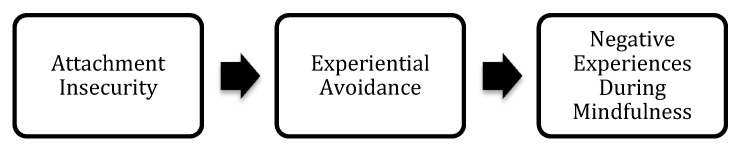
Hypothesized mediation model predicting the relationship between attachment insecurity (i.e., anxiety or avoidance) and negative experiences during mindfulness.

Finally, we collected data on participants’ actual engagement in and experience with mindfulness practices (i.e., meditation and yoga). We did not have hypotheses as to whether individuals higher in attachment insecurity would have engaged more or less in mindfulness practices. Due to the popularity of meditation and yoga in 2020 and many reasons for engaging in these activities beyond cultivating mindfulness (i.e., physical health, social and community engagement), we reasoned that individuals high and low in attachment insecurity would be just as likely to partake in these activities. However, we predicted that during engagement with mindfulness, individuals high in attachment insecurity would report more negative experiences (i.e., less positive feelings, more negative feelings, less focused attention, more judgment). We theorized that experiential avoidance, or the fear of engaging with one’s thoughts and feelings [74], would likely mediate this association. Attachment insecurity has been shown to be correlated with higher levels of experiential avoidance [74,77], thus potentially relating to more fear and struggles to remain in the present moment, especially if that present moment contains negative information (i.e., negative aspects of one’s environment, thoughts, or feelings). As mindfulness activities direct one’s attention to the present moment, individuals higher in attachment insecurity may report more negative experiences with these activities, and this may be partially explained due to higher levels of experiential avoidance (see hypothesis depicted visually in Figure 2).

Our hypotheses regarding the relationships among attachment, mindfulness, and relationship outcomes were explored using a brief electronic survey. This study was designed to be a starting point for further experimental research.

## 2. Materials and Methods

### 2.1. Participants

Three hundred and sixty individuals participated in this study (88.2% identifying as female, 11.5% male, 0.3% non-binary), with an average age of 21.6 years (ranging from 18 years to 60 years). The age distribution was skewed, with 90% of individuals younger than 23 years of age. Participants predominantly endorsed being Caucasian (72.1%), followed by East Asian (13.4%), South Asian (2.8%), Black (1.1%), Hispanic/Latinx (0.8%), and Indigenous (0.6%). 8.7% of the sample reported other ethnicities or multiple ethnicities, and 0.6% of the sample preferred not to respond. Measuring sexual orientation using the Kinsey Scale [80], 69.4% of participants reported being exclusively opposite gender-attracted, with variation across the seven-point spectrum to 1.4% reporting being exclusively same gender-attracted. 0.3% of the sample reported other sexual identities, 1.9% reported no sexual reactions or interest, and 0.3% preferred not to respond. Just over half of the participants were in romantic relationships (55%), with an average length of 2.5 years. This sample was collected from Facebook groups associated with our university, as well as Facebook groups for the greater community our university resides in. Participants who viewed the recruitment advertisements online self-selected to participate in the study by following an electronic link. Participants were compensated with entry into a monetary draw.

### 2.2. Measures and Procedure

Individuals provided their informed consent for inclusion before they participated in the study. Participants completed a twenty-minute online survey, providing demographic details, answering questionnaires about their attachment orientations, experiential avoidance, relationship satisfaction, mindfulness, rumination levels, and conflict styles, and then completed specific items about their experience with meditation and yoga. This procedure underwent a departmental and university-wide ethical review process approved by the Queen’s University General Research Ethics Board (approval code: 6027311).

Attachment orientations were assessed using the Experiences in Close Relationships-Revised Scale (ECR-R, [81]). This 36-item scale examines attachment anxiety (18 items, e.g., “I often worry that my partner doesn’t really love me”; Cronbach’s α = 0.929) and attachment avoidance (18 items, e.g., “I prefer not to be too close to romantic partners”; Cronbach’s α = 0.944) on a scale from 1 (Strongly Disagree) to 7 (Strongly Agree). All questionnaires used the same seven-point scale. Total scores were calculated using an average of all relevant items, reverse-scoring as indicated by measure protocols (i.e., a score of “7” is reverse-scored to a score of “1,” etc.).

The Acceptance and Action Questionnaire (AAQ-2, [82]) was used to examine experiential avoidance. This scale uses seven items to assess discomfort with one’s own internal experiences (e.g., “I’m afraid of my feelings”; Cronbach’s α = 0.889).

Relationship satisfaction was assessed for individuals in romantic relationships by adapting MacDonald and Ross’ [83] items (presented in Appendix A). Following an exploratory factor analysis, five items converged onto one factor, averaged to create an index of relationship satisfaction (Cronbach’s α = 0.848).

Relationship mindfulness was measured using the Relationship Mindfulness Measure (RMM, [84]). Five items examine mindfulness in relationships (e.g., “I have conversations with my partner without being really attentive”; Cronbach’s α = 0.780). This scale was only completed by individuals in relationships.

The Romantic Partner Conflict Scale (RPCS, [78]) was used to examine self-reported behavior during conflict between individuals in a romantic relationship. If participants were not in a relationship they reported on how they typically act in relationships. Thirty-nine items in total examine six subscales of conflict behaviors: compromise (14 items, e.g., “We try to find solutions that are acceptable to both of us”; Cronbach’s α = 0.947), avoidance (3 items, e.g., “I avoid conflict with my partner”; Cronbach’s α = 0.876), interactional reactivity (6 items, e.g., “When my partner and I disagree, we argue loudly”; Cronbach’s α = 0.867), separation (5 items, e.g., “When we experience conflict, we let each other cool off before discussing it further”; Cronbach’s α = 0.855), domination (6 items, e.g., “I try to take control when we argue”; Cronbach’s α = 0.874), and submission (5 items, e.g., “Sometimes I agree with my partner so the conflict will end”; Cronbach’s α = 0.920).

Rumination was assessed using the Ruminative Responses Scale (RRS, [85]). This scale has 22 items examining rumination (e.g., “When I feel down, sad, or depressed, I think about how alone I feel”; Cronbach’s α = 0.888). We created ten additional items examining rumination after conflict with one’s romantic partner (items in Appendix A). These items were averaged into a scale of rumination in relationships (Cronbach’s α = 0.830).

The Mindful Attention Awareness Scale was employed to measure mindfulness (MAAS, [23]). This scale uses fifteen items to examine dispositional levels of mindfulness (e.g., “I find myself doing things without paying attention”, reverse-scored; α = 0.875).

After completing personality questionnaires, participants were asked if they have ever participated in meditation or yoga. If they answered yes to either question, they answered several additional questions about their experiences with these practices (see questions in Appendix A). Following an exploratory factor analysis, the experience with meditation items converged onto four factors: attention to the present (2 items; Cronbach’s α = 0.809), nonattachment (3 items; Cronbach’s α = 0.730), positive feelings (4 items; Cronbach’s α = 0.797), and negative feelings (4 items; Cronbach’s α = 0.702). The experience with yoga items were entered into a separate factor analysis and emerged quite similarly, although not identical to the meditation items. Due to the many similarities and for reasons of consistency, the same factors were used for scale construction: attention to the present (2 items; Cronbach’s α = 0.685), nonattachment (3 items; Cronbach’s α = 0.828), positive feelings (4 items; Cronbach’s α = 0.745), and negative feelings (4 items; Cronbach’s α = 0.699).

Note that three additional personality questionnaires: the Big Five Inventory [86], the Rosenberg Self-Esteem Scale [87], and the Five Facet Mindfulness Questionnaire [88] were also included in this survey. As these questionnaires were not used to answer the research questions pertaining to this project, they are not discussed further.

## 3. Data Analytic Strategy

Scales were constructed by averaging all relevant variables, and then mean-centering the average scores. Dichotomous variables were also centered (e.g., relationship status was scored as either −1 or 1). Three factor analyses were conducted (i.e., for the relationship satisfaction items, experiences with meditation items, and experiences with yoga items) using principal axis factoring with a direct oblimin rotation (assuming intercorrelations between items). Items were retained if they loaded on one factor above 0.5. As stated in the Methods Section, the experiences with yoga items were organized into the same factors as the experiences with meditation items for consistency, as they formed a quite similar factor structure. Relationship satisfaction was negatively skewed, so scores were reflected and then a logarithmic function was applied to normalize the data. The same process occurred for the variable ‘paying attention to the present moment’ during yoga and meditation. We then examined demographics, correlations among the variables, and our mediation hypotheses using Hayes’ [89] Process Macro for SPSS. Model four was used for instances with one mediator (i.e., one mechanism predicting the relationship between two variables; similar to Figure 2 above), and model six was used for instances with two serial mediators (i.e., two mechanisms predicting the relationship between two variables, assumed to exert influence in a specific order; similar to Figure 1 above). For models with two serial mediators, both orders of the mediators were tested (i.e., one model with mindfulness entered first, followed by rumination, and the second model with the opposite order). As the general mindfulness and rumination scales were more reliable than the relationship-specific mindfulness and rumination scales, they were used as mediators in all analyses. However, note that we did examine the relationship-specific measures (i.e., relational mindfulness and rumination in relationships) as mediating variables in separate analyses. The results followed similar patterns to the generalized variables (i.e., general mindfulness and rumination), but not always to the level of significance. Thus, for the purposes of this article, we have reported models with the generalized variables.

## 4. Results

Data are available at: https://osf.io/pa8eh/?view_only=119809f2f3a545e7b5a6120170d48ea5.

### 4.1. Descriptive Statistics and Correlations

Descriptive statistics and correlations among the variables are presented in Table 1, Table 2, Table 3 and Table 4. As hypothesized, there were significant positive correlations between attachment insecurity (i.e., both attachment anxiety and avoidance) and experiential avoidance and rumination (both for general and relationship-specific measures, all *p* < 0.001, see Table 2 for specific correlation coefficients). Additionally, there were significant negative correlations between attachment insecurity and mindfulness (both for general and relationship-specific measures, all *p* < 0.05, see Table 2), as well as with relationship satisfaction (both *p* < 0.001, see Table 3). Attachment insecurity was negatively associated with compromise, and positively associated with avoidance, interactional reactivity, separation, domination, and submission (all *p* < 0.05, see Table 3). Attachment insecurity was also associated with negative feelings during meditation and yoga (all *p* < 0.01, see Table 4). Attachment avoidance specifically was associated with less attention to the present during yoga (*r* = −0.282, *p* < 0.01). Contrary to our hypotheses, attachment anxiety and avoidance were not associated with less positive experiences during mindfulness activities or difficulties with non-attachment, and attachment anxiety was not associated with less attention to the present (all *p* > 0.05, see Table 4).

### 4.2. Mediation Analyses with Conflict Outcomes

We then tested serial mediation models of whether mindfulness (measured using the MAAS, [23]) and rumination (measured using the RRS, [85]) mediated the relationship between attachment orientations and conflict styles. Separate serial mediations were conducted for all six conflict styles as outcome variables, with anxiety and avoidance in different analyses. We conducted these mediations with both orderings of the mediating variables (i.e., mindfulness and then rumination, rumination and then mindfulness). The results of the mediation analyses are presented descriptively in Table 5 and detailed with statistical values afterwards.

#### 4.2.1. Serial Mediations with Attachment Anxiety as the Predictor

Note that for all mediation analyses described below, we first examined the serial mediations with the order of mediators: mindfulness, and then rumination. These are the results written in full. We then tested the opposite ordering of mediators: rumination, and then mindfulness. The indirect effect for models with this ordering is communicated at the end of each paragraph. The direct effect captures the relationship between the independent variable and the outcome variable without the mediating variables accounted for, whereas the indirect effect captures the relationship between the independent variable and the outcome variable with the mediating variables incorporated into the model (i.e., factored in as mechanisms explaining variance in the outcome variable). To have a significant mediation model, the indirect effect must be significant (i.e., the confidence interval must not cross zero). We have also included a measure of effect size (Cohen’s *f*^2^, calculated from *R*^2^) [90,91]. Cohen’s *f*^2^ is a standardized measure of effect size, or the strength of the relationship between two variables. It is generally interpreted as *f*^2^ ≥ 0.02 represents a small effect, *f*^2^ ≥ 0.15 represents a medium effect, and *f*^2^ ≥ 0.35 represents a large effect [90]; however, these categorical distinctions are somewhat arbitrary.

##### Compromise

Attachment anxiety was associated with less mindfulness (*b* = −0.35, *p* < 0.001) and more rumination (*b* = 0.30, *p* < 0.001). Mindfulness was associated with less rumination (*b* = −0.38, *p* < 0.001). Attachment anxiety predicted less compromise through a direct effect (*b* = −0.22, *p* < 0.001), and also indirectly through the serial mediators of mindfulness and rumination (95% confidence interval {*CI*}*:* 0.0084, 0.0500). The model predicting compromise with attachment anxiety, mindfulness, and rumination was determined to have a medium effect size (*R*^2^ = 0.13, *f*^2^ = 0.15). By switching the mediators’ order (i.e., rumination and then mindfulness), a similar pattern emerged, with a significant indirect effect (95% *CI*: −0.0744, −0.0173).

##### Avoidance

Attachment anxiety was associated with less mindfulness (*b* = −0.35, *p* < 0.001) and more rumination (*b* = 0.30, *p* < 0.001). Mindfulness was associated with less rumination (*b* = −0.38, *p* < 0.001). Attachment anxiety was not associated with avoidance during conflict through a direct effect (*b* = 0.11, *p* > 0.05), although the indirect effect through the serial mediators of mindfulness and rumination was indeed significant (95% *CI*: 0.0136, 0.0870). The model predicting avoidance with attachment anxiety, mindfulness, and rumination was determined to have a small effect size (*R*^2^ = 0.07, *f*^2^ = 0.07). By switching the mediators’ order (i.e., rumination and then mindfulness), the indirect effect was not significant (i.e., confidence interval crossed zero).

##### Interactional Reactivity

Attachment anxiety was associated with less mindfulness (*b* = −0.36, *p* < 0.001) and more rumination (*b* = 0.30, *p* < 0.001). Mindfulness was associated with less rumination (*b* = −0.37, *p* < 0.001). Attachment anxiety predicted greater interactional reactivity through a direct effect (*b* = 0.51, *p* < 0.001), and also indirectly through the serial mediators of mindfulness and rumination (95% *CI*: −0.0718, −0.0127; confidence interval does not cross zero, indicating statistical significance). The model predicting interactional reactivity with attachment anxiety, mindfulness, and rumination was determined to have a medium effect size (*R*^2^ = 0.25, *f*^2^ = 0.33). By switching the mediators’ order (i.e., rumination and then mindfulness), a similar pattern emerged, with a significant indirect effect (95% *CI*: 0.0189, 0.0890).

##### Separation

Attachment anxiety was associated with less mindfulness (*b* = −0.35, *p* < 0.001) and more rumination (*b* = 0.30, *p* < 0.001). Mindfulness was associated with less rumination (*b* = −0.38, *p* < 0.001). Attachment anxiety was not associated with separation during conflict to our cutoff for significance (*b* = 0.15, *p* = 0.054, marginal effect); however, the indirect effect was indeed significant through the serial mediators of mindfulness and rumination (95% *CI*: 0.0127, 0.0732). The model predicting separation with attachment anxiety, mindfulness, and rumination was determined to have a small effect size (*R*^2^ = 0.09, *f*^2^ = 0.10). By switching the mediators’ order (i.e., rumination and then mindfulness), the indirect effect was not significant (i.e., confidence interval crossed zero).

##### Domination

Attachment anxiety was associated with less mindfulness (*b* = −0.35, *p* < 0.001) and more rumination (*b* = 0.30, *p* < 0.001). Mindfulness was associated with less rumination (*b* = −0.38, *p* < 0.001). Attachment anxiety predicted more domination through a direct effect (*b* = 0.19, *p* = 0.011), and also indirectly through the serial mediators of mindfulness and rumination (95% *CI*: −0.0586, −0.0024). The model predicting domination with attachment anxiety, mindfulness, and rumination was determined to have a small effect size (*R*^2^ = 0.11, *f*^2^ = 0.13). By switching the mediators’ order (i.e., rumination and then mindfulness), a similar pattern emerged, with a significant indirect effect (95% *CI*: 0.0301, 0.1165).

##### Submission

Attachment anxiety was associated with less mindfulness (*b* = −0.35, *p* < 0.001) and more rumination (*b* = 0.30, *p* < 0.001). Mindfulness was associated with less rumination (*b* = −0.38, *p* < 0.001). Attachment anxiety predicted more submission through a direct effect (*b* = 0.24, *p* = 0.001), and also indirectly through the serial mediators of mindfulness and rumination (95% *CI*: 0.0102, 0.0692). The model predicting submission with attachment anxiety, mindfulness, and rumination was determined to have a medium effect size (*R*^2^ = 0.20, *f*^2^ = 0.25). By switching the mediators’ order (i.e., rumination and then mindfulness), a similar pattern emerged, with a significant indirect effect (95% *CI*: 0.0024, 0.0679).

#### 4.2.2. Serial Mediations with Attachment Avoidance as the Predictor

##### Compromise

Attachment avoidance was associated with less mindfulness (*b* = −0.30, *p* < 0.001), but not associated with rumination (*b* = 0.07, *p* > 0.05). Mindfulness was associated with less rumination (*b* = −0.48, *p* < 0.001). Attachment avoidance predicted less compromise through a direct effect (*b* = −0.36, *p* < 0.001), and also indirectly through the serial mediators of mindfulness and rumination (95% *CI*: 0.0034, 0.0397). The model predicting compromise with attachment avoidance, mindfulness, and rumination was determined to have a medium effect size (*R*^2^ = 0.24, *f*^2^ = 0.31). By switching the mediators’ order (i.e., rumination and then mindfulness), this indirect effect was also significant (95% *CI*: −0.0376, −0.0046).

##### Avoidance

Attachment avoidance was associated with less mindfulness (*b* = −0.30, *p* < 0.001), but not associated with rumination (*b* = 0.07, *p* > 0.05). Mindfulness was associated with less rumination (*b* = −0.48, *p* < 0.001). Attachment avoidance was not associated with avoidance during conflict to our cutoff for significance (*b* = 0.16, *p* < 0.059, marginal effect); however, the indirect effect was indeed significant through the serial mediators of mindfulness and rumination (95% *CI*: 0.0203, 0.1012). The model predicting avoidance with attachment avoidance, mindfulness, and rumination was determined to have a small effect size (*R*^2^ = 0.08, *f*^2^ = 0.08). By switching the mediators’ order (i.e., rumination and then mindfulness), the indirect effect was not significant (i.e., confidence interval crossed zero).

##### Interactional Reactivity

Attachment avoidance was associated with less mindfulness (*b* = −0.30, *p* < 0.001), but not associated with rumination (*b* = 0.06, *p* > 0.05). Attachment avoidance predicted greater interactional reactivity through a direct effect (*b* = 0.24, *p* < 0.001); however, the indirect effect was not significant (i.e., confidence interval crossed zero). The model predicting interactional reactivity with attachment avoidance, mindfulness, and rumination was determined to have a medium effect size (*R*^2^ = 0.13, *f*^2^ = 0.15). By switching the mediators’ order (i.e., rumination and then mindfulness), this indirect effect was indeed significant (95% *CI*: 0.0106, 0.0611).

##### Separation

Attachment avoidance was associated with less mindfulness (*b* = −0.30, *p* < 0.001), but not associated with rumination (*b* = 0.07, *p* > 0.05). Mindfulness was associated with less rumination (*b* = −0.48, *p* < 0.001). Attachment avoidance predicted more separation through a direct effect (*b* = 0.27, *p* < 0.001), and also indirectly through the serial mediators of mindfulness and rumination (95% *CI*: 0.0169, 0.0813). The model predicting separation with attachment avoidance, mindfulness, and rumination was determined to have a small effect size (*R*^2^ = 0.13, *f*^2^ = 0.14). By switching the mediators’ order (i.e., rumination and then mindfulness), the indirect effect was not significant (i.e., confidence interval crossed zero).

##### Domination

Attachment avoidance was associated with less mindfulness (*b* = −0.30, *p* < 0.001), but not associated with rumination (*b* = 0.07, *p* > 0.05). Mindfulness was associated with less rumination (*b* = −0.48, *p* < 0.001). Attachment avoidance predicted more domination through a direct effect (*b* = 0.22, *p* = 0.002); however, the indirect effect was not significant (i.e., confidence interval crossed zero). The model predicting domination with attachment avoidance, mindfulness, and rumination was determined to have a small effect size (*R*^2^ = 0.12, *f*^2^ = 0.14). By switching the mediators’ order (i.e., rumination and then mindfulness), this indirect effect was indeed significant (95% *CI*: 0.0120, 0.0679).

##### Submission

Attachment avoidance was associated with less mindfulness (*b* = −0.30, *p* < 0.001), but not associated with rumination (*b* = 0.07, *p* > 0.05). Mindfulness was associated with less rumination (*b* = −0.48, *p* < 0.001). Attachment avoidance was not associated with submission through a direct effect (*b* = 0.07, *p* > 0.05); however, the indirect effect was indeed significant through the serial mediators of mindfulness and rumination (95% *CI*: 0.0231, 0.0943). The model predicting submission with attachment avoidance, mindfulness, and rumination was determined to have a medium effect size (*R*^2^ = 0.16, *f*^2^ = 0.20). By switching the mediators’ order (i.e., rumination and then mindfulness), this indirect effect was also significant (95% *CI*: 0.0030, 0.0462).

### 4.3. Mediation Analyses with Relationship Satisfaction

We also examined relationship satisfaction as an outcome for these serial mediation models. None of the mediation models were significant for this outcome (i.e., for attachment anxiety or avoidance, with either order of mediating variables).

### 4.4. Mediation Analyses Examining Attachment Insecurity, Experiential Avoidance, and Experiences During Mindfulness

As a final set of analyses, we examined whether experiential avoidance mediated the relationship between attachment orientations and negative feelings during meditation and yoga. All four mediation models were indeed significant.

Attachment anxiety was associated with higher experiential avoidance (*b* = 0.62, *p* < 0.001). Experiential avoidance predicted more negative feelings during meditation (*b* = 0.24, *p* = 0.002). Attachment anxiety did not predict negative experiences during meditation directly (*b* = 0.10, *p* > 0.05); however, the indirect effect through experiential avoidance was indeed significant (95% *CI*: 0.0475, 0.2691). The model predicting negative experiences with meditation with attachment anxiety and experiential avoidance was determined to have a small effect size (*R*^2^ = 0.11, *f*^2^ = 0.12).

Attachment anxiety was associated with higher experiential avoidance (*b* = 0.60, *p* < 0.001). Experiential avoidance predicted more negative feelings during yoga (*b* = 0.32, *p* < 0.001). Attachment anxiety did not predict negative experiences during yoga directly (*b* = 0.05, *p* > 0.05); however, the indirect effect through experiential avoidance was indeed significant (95% *CI*: 0.0988, 0.3259). The model predicting negative experiences with yoga with attachment anxiety and experiential avoidance was determined to have a medium effect size (*R*^2^ = 0.16, *f*^2^ = 0.19).

Attachment avoidance was associated with higher experiential avoidance (*b* = 0.26, *p* = 0.010). Experiential avoidance predicted more negative feelings during meditation (*b* = 0.25, *p* < 0.001). Attachment avoidance was associated with negative experiences during meditation directly (*b* = 0.18, *p* = 0.034), and also indirectly through experiential avoidance (95% *CI*: 0.0127, 0.1509). The model predicting negative experiences with meditation with attachment avoidance and experiential avoidance was determined to have a medium effect size (*R*^2^ = 0.13, *f*^2^ = 0.15).

Attachment avoidance was associated with higher experiential avoidance (*b* = 0.31, *p* = 0.002). Experiential avoidance predicted more negative feelings during yoga (*b* = 0.31, *p* < 0.001). Attachment avoidance was associated with negative experiences during yoga directly (*b* = 0.17, *p* = 0.047), and also indirectly through experiential avoidance (95% *CI*: 0.0257, 0.1911). The model predicting negative experiences with yoga with attachment avoidance and experiential avoidance was determined to have a medium effect size (*R*^2^ = 0.18, *f*^2^ = 0.22).

## 5. Discussion

Replicating past literature, attachment insecurity was indeed related to lower levels of mindfulness (e.g., reference [68]), increased rumination (e.g., reference [6]), and increased experiential avoidance (e.g., reference [76]). Attachment insecurity was also related to decreased relationship satisfaction and all six conflict styles (less compromise, and more interactional reactivity, avoidance, separation, domination, and submission). This replicates past patterns of attachment insecurity predicting more negative conflict behaviors [14,15,16,17]. Mindfulness and rumination together mediated the relationship between attachment insecurity and conflict styles for most of these models. This finding suggests that mindfulness and rumination are mechanisms explaining part of the relationship between attachment insecurity and negative conflict behavior. For attachment anxiety, all models were significant when the mediators were ordered with mindfulness first, and then rumination. Switching the order of mediators, only models with the outcomes avoidance and separation were not significant. For attachment avoidance, there was greater variability in which ordering of mediators was related to a significant serial mediation. For the outcomes compromise and submission, both orderings were significant. For the outcomes avoidance and separation, mindfulness had to be ordered first for a significant mediation effect, while for the outcomes interactional reactivity and domination, rumination had to be ordered first for a significant mediation effect. As the relationships between mindfulness and rumination are likely bidirectional, this demonstrates the importance of testing both ordering of mediating variables in serial mediation models.

Despite differences in ordering, we have found evidence that mindfulness and rumination are mechanisms related to the relationship between attachment insecurity and negative conflict behavior. These may be important targets for therapeutic intervention for couples in conflict. By increasing attention to the present moment and reducing bias and judgment (two important components of mindfulness [24]), individuals high in attachment insecurity may be better able to regulate their emotions, reduce their rumination, and engage in relationship-promoting behavior.

As noted in the Data Analytic Strategy we conducted a separate series of mediation models with relationship-specific rumination and mindfulness entered as mediating variables. These analyses produced a similar pattern of results as the models with the general mindfulness and rumination measures; however, not always to a level of statistical significance. As only individuals in romantic relationships completed the Relationship Mindfulness Measure [84], the statistical models examining relationship-specific variables had smaller sample sizes than the models with generalized mediating variables (i.e., with the MAAS measure of mindfulness that all participants completed [23]). We conducted post-hoc power analyses examining the observed statistical power for our serial mediation models. Observed power for the model of attachment anxiety predicting compromise with the generalized mediating variables was extremely high (>0.999). Observed power was also extremely high for the model with the relationship-specific mediating variables (0.992). This was also true substituting attachment avoidance as the predictor rather than attachment anxiety (observed power of >0.999 and >0.999 for the models with generalized and relationship-specific mediating variables, respectively). Thus, statistical power was high in our analyses regardless of whether generalized or relationship-specific variables were used, and we should have been able to observe true effects should they exist in our sample (i.e., type 2 errors were unlikely). Accordingly, the generalized variables and relational variables may capture slightly different constructs and may relate to conflict behaviors and attachment orientations in slightly different ways. To simplify our results, we only communicated models with the generalized variables, as the effects were more robust. We encourage future researchers to examine the relationship-specific mindfulness and rumination variables, and the unique effects they may relate to.

With relationship satisfaction as the outcome in our serial mediation models, we did not observe significant effects. This is despite attachment insecurity significantly correlating with decreased levels of relationship satisfaction. We wonder if other variables may better account for the relationship between attachment insecurity and relationship satisfaction, rather than mindfulness and rumination (e.g., trust for individuals high in attachment anxiety). Alternatively, there may be key moderators missing from this serial mediation effect. For example, mindfulness and rumination may mediate the link between attachment insecurity and relationship satisfaction, but only for couples that have frequent conflict. When conflict is more frequent, low levels of mindfulness and high levels of rumination may exert a greater influence on interpersonal behavior, and thus impact relationship satisfaction to a greater extent.

Finally, we found that attachment anxiety and avoidance were correlated with more negative feelings during yoga and mindfulness (i.e., feeling frustrated, competitive, and bored). This relationship was mediated by experiential avoidance, or the fear of engaging with one’s thoughts and feelings. It follows that fearing engagement with one’s internal experiences would predict greater difficulty with mindfulness activities, and more negative feelings towards them. For this reason, we expected that individuals high in attachment insecurity would also report less positive feelings during mindfulness, less attention to the present, and less non-judgment (otherwise stated as more judgmental thoughts, attachment, and bias). These relationships were not substantiated. We wonder if reporting experiences with mindfulness on an online questionnaire may be too removed from the experiences to be able to accurately measure them. Perhaps negative experiences during mindfulness are memorable and able to be more accurately reported, but challenges with attention and non-judgment may need to be measured during actual mindfulness activities. Experimental research with in-the-moment measurements would be able to clarify this result.

Overall, the finding that individuals high in attachment anxiety and avoidance are more likely to report negative feelings during mindfulness is meaningful and novel. If mindfulness experiences are negative, individuals may be less motivated to continue to engage in these practices. The goal of mindfulness is not typically to ensure a positive experience (rather it is about being present and aware of all sensations, regardless of if they are good or bad); however, negative experiences without training and support may deter individuals high in attachment insecurity from engaging in these practices in the future. As mindfulness is a mechanism relating attachment insecurity to negative conflict behaviors, it could be an important target for improving relationship functioning for these individuals. Thus, targeting experiential avoidance may need to occur first, to then see greater engagement in mindfulness and altered behavior during relational conflict. Through treatment targeted towards reducing fears of negative internal and external events, and improving the capacity to sit with and then cope with these negative experiences (e.g., in Acceptance and Commitment Therapy and Dialectical Behavior Therapy, [45,46]), individuals high in attachment insecurity may be more motivated and able to engage in mindfulness. This would thus relate to reductions in rumination and more positive engagement during relational conflict. Experiential avoidance appears to be a helpful starting point for intervention, with potentially a chain of positive interpersonal and relational improvements related to increased mindfulness. With these improvements, there may be benefits to both the individual’s mental health and emotional experience, as well as to the relationship (i.e., better communication, more sensitive and responsive behavior during conflict). This could potentially relate to enhanced security for both members of the couple, reducing anxiety and avoidance over time. We acknowledge that these results are preliminary, and we hope that they may serve as a starting point for further experimental exploration. More research is needed to determine the causal ordering of the relationship between these variables, and relevant boundary conditions of individual and situational factors that may influence these effects. We are currently exploring mindfulness manipulations in our laboratory to examine effects on attachment insecurity, rumination, and relational outcomes, and encourage others to continue this work as well.

There are a number of limitations to this study. These data were collected using an online, self-report questionnaire, and individuals may not be able to accurately report certain aspects of their relationship or experience with mindfulness. Further, the most significant limitation of this study is the homogeneity of the sample. We recruited widely across community groups from our university and greater community, but as participants self-selected to participate in our study by following an online survey link, our sample was especially skewed to be young, white, heterosexual, and female. These results may not accurately reflect the experiences of other individuals outside of this identity. We are continuing research to better understand the generalizability of our findings.

## 6. Conclusions

This study serves as a starting point to uncover specifically how mindfulness (or a lack thereof) is related to behavior during conflict in romantic relationships. This research provides a unique contribution to the literature, exploring how mindfulness and attachment orientations relate to specific behaviors during conflict in relationships. To our knowledge, no other research has discovered distinct pathways between these cognitive, affective, and interpersonal constructs. Individuals high in attachment insecurity appear to report lower levels of mindfulness, increased rates of rumination, and more negative conflict behaviors. Although these relationships are correlational, our research suggests that increasing mindfulness may be related to improved personal and relational outcomes, specifically relating to reductions in rumination and increases in more prosocial conflict behaviors (e.g., compromise). Further, our research suggests that mindfulness practices may be associated with negative feelings for individuals high in attachment insecurity. This finding indicates a potential barrier for individuals high in attachment insecurity to engage in mindfulness as a coping strategy for improving their romantic relationship. Thus, reducing experiential avoidance may be an important first step in support individuals high in attachment insecurity to engage with mindfulness practices.

These findings have implications for developing theoretical models of the processes linking attachment orientations to conflict in romantic relationships, and also have real-world applications for supporting couples in conflict. Therapists working with couples in conflict are encouraged to target increasing mindfulness and reducing rumination, as both factors are associated with more prosocial conflict-management strategies. Further, practitioners are encouraged to consider how individuals high in attachment insecurity may require greater levels of support in engaging in mindfulness-building activities, potentially working to reduce experiential avoidance first in therapy, before proceeding to mindfulness-based treatment targets. Future research that assesses causality among these variables will be valuable in clarifying the directionality of these relationships, and in designing evidence-based strategies for therapy.

## Figures and Tables

**Table 1:** Descriptive statistics for untransformed study variables.

Variable	Mean	Standard Deviation
Attachment Anxiety	3.73	1.11
Attachment Avoidance	3.05	1.07
Experiential Avoidance	4.15	1.36
General Rumination	4.72	1.01
Rumination in Relationships	4.45	1.00
General Mindfulness	3.89	1.01
Mindfulness in Relationships	4.63	1.23
Relationship Satisfaction	5.84	0.97
Compromise	5.32	0.90
Avoidance	4.55	1.40
Interactional Reactivity	2.52	1.19
Separation	3.94	1.22
Domination	3.31	1.19
Submission	3.63	1.22
Meditation: Attention to Present	4.87	1.23
Meditation: Non-Attachment	5.93	1.08
Meditation: Positive Feelings	4.63	1.14
Meditation: Negative Feelings	3.34	1.18
Yoga: Attention to Present	4.55	1.38
Yoga: Non-Attachment	5.98	0.90
Yoga: Positive Feelings	5.10	0.97
Yoga: Negative Feelings	3.19	1.15

**Table 2:** Correlations among attachment orientations, experiential avoidance, rumination, and mindfulness variables.

Variable	1.	2.	3.	4.	5.	6.
1. Attachment Anxiety						
2. Attachment Avoidance	0.392 ***					
3. Experiential Avoidance	0.537 ***	0.240 ***				
4. General Rumination	0.487 ***	0.224 ***	0.671 ***			
5. Relationship Rumination	0.552 ***	0.220 ***	0.429 ***	0.567 ***		
6. General Mindfulness	−0.389 ***	−0.320 ***	−0.564 ***	−0.504 ***	−0.288 ***	
7. Relationship Mindfulness	−0.358 ***	−0.202 *	−0.446 ***	−0.372 ***	−0.305 **	0.491 ***

* *p* < 0.05, ** *p* < 0.01, *** *p* < 0.001.

**Table 3:** Correlations among attachment orientations, relationship satisfaction, and conflict styles.

Variable	1.	2.	3.	4.	5.	6.	7.	8.
1. Attachment Anxiety								
2. Attachment Avoidance	0.392 ***							
3. Relationship Satisfaction	−0.427 ***	−0.409 ***						
Conflict Styles								
4. Compromise	−0.260 ***	−0.473 ***	0.362 ***					
5. Avoidance	0.172 **	0.150 *	−0.039	0.022				
6. Interactional Reactivity	0.442 ***	0.291 ***	−0.427 ***	−0.489 ***	−0.048			
7. Separation	0.232 ***	0.290 ***	−0.245 **	−0.159 **	0.155 *	0.303 ***		
8. Domination	0.229 ***	0.267 ***	−0.321 ***	−0.334 ***	−0.021	0.478 ***	0.370 ***	
9. Submission	0.370 ***	0.177 **	−0.244 **	−0.266 ***	0.294 ***	0.344 ***	0.285 ***	0.149 *

* *p* < 0.05, ** *p* < 0.01, *** *p* < 0.001.

**Table 4:** Correlations among attachment orientations, experience with meditation, and experience with yoga variables.

Variable	1.	2.	3.	4.	5.	6.	7.	8.	9.
1. Attachment Anxiety									
2. Attachment Avoidance	0.392 ***								
Meditation Variables									
3. Attention to Present	−0.044	−0.089							
4. Non-Attachment	0.102	−0.028	0.276 ***						
5. Positive Feelings	−0.008	−0.044	0.200 *	0.485 ***					
6. Negative Feelings	0.224 **	0.223 **	−0.184 *	−0.047	−0.177 *				
Yoga Variables									
7. Attention to Present	0.000	−0.282 **	0.506 ***	0.256 **	0.384 ***	−0.206 *			
8. Non-Attachment	0.088	0.022	0.108	0.444 ***	0.467 ***	−0.092	0.308 ***		
9. Positive Feelings	−0.010	−0.090	0.314 **	0.309 **	0.595 ***	−0.175	0.508 ***	0.520 ***	
10. Negative Feelings	0.239 **	0.245 **	−0.118	−0.054	−0.053	0.496 ***	−0.395 ***	−0.131	−0.308 ***

* *p* < 0.05, ** *p* < 0.01, *** *p* < 0.001.

**Table 5:** Summary of serial mediation analyses.

Conflict Style (Outcome Variable)	Attachment Anxiety as Predictor		Attachment Avoidance as Predictor	
	Mediator Order: 1. Mindfulness 2. Rumination	Mediator Order: 1. Rumination 2. Mindfulness	Mediator Order: 1. Mindfulness 2. Rumination	Mediator Order: 1. Rumination 2. Mindfulness
Compromise	Significant	Significant	Significant	Significant
Avoidance	Significant	Non-significant	Significant	Non-significant
Interactional Reactivity	Significant	Significant	Non-significant	Significant
Separation	Significant	Non-significant	Significant	Non-significant
Domination	Significant	Significant	Non-significant	Significant
Submission	Significant	Significant	Significant	Significant

## Appendix A

### Self-Created Study Materials

#### Relationship Items

Adapted from MacDonald and Ross’ [83] protocol.

How serious is your relationship?
How satisfied are you with your relationship overall? *
How satisfied are you with the romantic aspects of your relationship? *
How satisfied are you with the physical and/or sexual aspects of your relationship? *
How satisfied are you with the emotional and/or supportive aspects of your relationship? *
How satisfied are you with the amount of fun you have in your relationship? *
How in love are you?
To what extent do you feel like you are growing together?

Note: starred items (*) were averaged to create an index of relationship satisfaction following a factor analysis.

#### Rumination in Relationships Items

Adapted from the Ruminative Responses Scale (RRS, [85]).

Think about conflict with your romantic partner. This can be a past partner or current relationship. After most arguments, rate the degree to which you think about the following things:

Your relationship.
How to improve your relationship.
Whether your partner was mad.
What you did to deserve this.
Why you always act this way.
Why your partner always acts this way.
Why the situation hadn’t gone better.
Why you have problems other people don’t have.
Why you can’t handle things better.
Why your partner can’t handle things better.

Note: all items were averaged to create an index of rumination in relationships.

#### Experience with Meditation and Yoga Items

Have you ever meditated/practiced yoga before? (Asked separately; if “yes”, the following items were asked):

How long have you been meditating/practicing yoga?
How often do you meditate/practice yoga?
Which resources have you used to learn about meditating/yoga? (select all that apply: classes, workshops, books, podcasts, movies/documentaries, a teacher, a friend, a phone application, other).
Do you practice on your own or with a guide/teacher (i.e., in a class, with a phone application)?
What type of meditations do you practice? (select all that apply: breath awareness, body scanning, concentration, grounding, visualization, insight, gratitude, loving kindness, mindful attention, mindful listening, noting, progressive muscle relaxation, other, I’m not sure).
What type of yoga do you practice? (select all that apply: vinyasa, power, hatha, hot, yin, restorative, other, I’m not sure).
Why do you practice yoga/meditation? (rate the following reasons and/or provide your own: attention/memory, relaxation, mental health, physical health, community, other).

Please rate the extent you agree with the following:

When I do yoga/meditate I…

1. Focus on the present.
2. Focus on my breath.
3. Focus on my body.
4. Practice being non-judgmental.
5. Practice letting go.
6. Practice non-attachment.
7. Get frustrated when my mind wanders.
8. Experience positive feelings more strongly than if I wasn’t meditating/doing yoga.
9. Experience negative feelings more strongly than if I wasn’t meditating/doing yoga.
10. Feel energized.
11. Feel happy.
12. Feel calm.
13. Feel frustrated.
14. Feel competitive.
15. Feel bored.
16. Feel determined.

Note: following a factor analysis, items 2 and 3 were averaged for a scale of attention to the present, items 4, 5, and 6 were averaged for a scale of nonattachment, items 8, 10, 11, and 16 were averaged for a scale of positive feelings, and items 9, 13, 14, and 15 were averaged for a scale of negative feelings. Items 1, 7, and 12 did not load sufficiently on any of the factors and were not included in scale construction.
